# Evaluating the Performance of Gravity-Driven Membrane Filtration for Waterborne Pathogen Removal and Public Health Protection

**DOI:** 10.1007/s12560-025-09655-1

**Published:** 2025-07-14

**Authors:** Chaojie Li

**Affiliations:** Department Surface Waters Research & Management, Eawag, Swiss Federal Institute of Aquatic Science and Technology, Seestrasse 79, 6047 Kastanienbaum, Switzerland

**Keywords:** Membrane filtration, Wastewater, Microbial risk assessment, Enteric virus

## Abstract

Waterborne pathogens pose a serious threat to public health, emphasizing the need for reliable and efficient water treatment technologies. Wastewater treatment plants employ a range of processes to reduce microbial contamination, with membrane filtration emerging as a promising solution due to its ability to physically remove pathogens without the production of harmful chemical by-products. This study investigates the effectiveness of a gravity-driven membrane (GDM) filtration system for pathogen removal from wastewater and evaluates the associated public health risks with and without treatment. A quantitative microbial risk assessment model was employed to estimate infection probabilities for various waterborne pathogens. The results demonstrated a significant decrease in pathogen concentrations following treatment, with up to a 10^4^-fold reduction in norovirus infection risk. Three critical factors influencing membrane performance were identified: membrane integrity, pore size characteristics, and membrane fouling. Maintaining membrane integrity was found to be essential for ensuring consistent pathogen removal. While nominal pore size is commonly used to predict rejection efficiency, the overall pore size distribution was found to have a greater influence on virus retention. Additionally, although membrane fouling is often considered detrimental, it was shown to enhance virus removal by up to two orders of magnitude. These findings underscore the potential of GDM systems for effective virus removal and highlight the importance of proper membrane design, maintenance, and monitoring in ensuring long-term operational efficiency and maximizing public health protection in wastewater treatment applications.

**Supplementary Information:**

The online version contains supplementary material available at 10.1007/s12560-025-09655-1.

## Introduction

Waterborne pathogens are frequently present in wastewater effluents, which are discharged into receiving water bodies, such as rivers, lakes, and wetlands. These pathogens pose significant health risks to individuals exposed to contaminated water, as seemingly clean water can still harbor relatively high pathogen concentrations (WHO, 2022). For instance, while wastewater treatment plant (WWTP) processes are designed to inactivate or remove enteric viruses, the reduction in virus concentrations is often less pronounced compared to other parameters (Kokkinos et al., 2015). Conventional WWTPs employing primary and secondary treatment processes typically achieve a 1–3 log₁₀ reduction (log removal) in enteric virus concentrations (Gibson, 2014; Verbyla & Mihelcic, 2015). However, this reduction may not be sufficient to ensure low infection risks for people exposed to treated wastewater, as efficiency varies by treatment process and virus type (Kitajima et al., 2018). To enhance effluent water quality, membrane technologies are increasingly integrated into wastewater treatment processes (Pronk et al., 2019). Gravity-driven ultrafiltration membrane (GDM) systems have gained traction for treating wastewater and greywater due to their low energy consumption, minimal maintenance, and suitability for decentralized applications (Smith et al., 2012). Ultrafiltration membranes, with pore sizes ranging from approximately 5 to 50 nm, can act as effective barriers to bacteria and, in some cases, viruses. However, maintaining membrane integrity is critical to ensuring pathogen removal. If membrane integrity is compromised, pathogens can pass through, degrading the permeate quality (Pype et al., 2016). A sensitive and effective membrane integrity monitoring technique is essential (Bivins et al., 2020).

Various membrane integrity tests have been developed, broadly categorized into direct and indirect methods (Pype et al., 2016). Direct methods, such as the bubble point test, pressure decay test (PDT), diffusive airflow (DAF) test, acoustic sensor test, and binary gas integrity test, directly assess the membrane or its modules (Guo et al., 2010). While highly sensitive, these methods cannot be conducted online and do not provide real-time information about permeate quality. Indirect methods, including particle counting, particle monitoring, turbidity monitoring, and surrogate challenge tests, continuously monitor parameters in the permeate (Persidsky & Baillie, 1977). Although less sensitive, they offer operational continuity. Surrogate challenge tests combine the advantages of both approaches by providing high sensitivity, online applicability, and a direct relationship between the test results and pathogen removal efficiency. Identifying an appropriate surrogate is key to this approach—one that is sensitive, representative of pathogens, non-destructive, non-toxic, and cost-effective.

In addition to membrane integrity, pore size and uniformity are critical factors in determining pathogen removal efficiency (Košutić & Kunst, 2002). Since waterborne pathogen sizes vary significantly, selecting an appropriate membrane type is essential. However, smaller pore sizes in GDM systems can result in reduced permeate flux, necessitating a balance between removal efficiency and filtration performance.

Membrane fouling, which leads to the formation of a biofilm layer, is another important factor affecting GDM system performance (Derlon et al., 2012). While fouling typically reduces flux and is considered undesirable, in GDM systems, biofilms can act as a “secondary membrane,” enhancing pathogen removal. A typical GDM system with ultrafiltration membranes achieves approximately a 6-log₁₀ reduction in bacteria and a 3-log₁₀ reduction in viruses, and the log removal value (LRV) is defined by (Branch et al., 2021)

1 $${\text{LRV}}\; = \;\log \frac{{C_{{\text{f}}} }}{{C_{{\text{p}}} }}$$

where *C*_f_ is the concentration in the feed and *C*_p_ is the concentration in the permeate. But when membranes are compromised, the impact of breaches can be calculated as follows:

2 $$\Delta {\text{LRV}}\; = \;{\text{LRV}}_{1} \; - \;\log_{10} \left( {\frac{{C_{{\text{f}}} V_{{\text{T}}} }}{{C_{{\text{P}}} V_{{\text{P}}} \; + \;C_{{\text{f}}} V_{{\text{d}}} }}} \right)$$

where LRV_1_ is the LRV of the undamaged part of the membrane, *V*_P_ is the feed volume passing through this undamaged part, *V*_d_ is the feed volume passing through the impaired part of the membrane, and *V*_T_ is the total feed volume that filtrates through the whole membrane.

In this study, we collected influent pathogen concentrations from eight WWTPs over three continents and chose norovirus as our research target and used a quantitative microbial risk assessment (QMRA) to quantify potential health risks associated with contact with pathogen-contaminated water. Norovirus was selected as the target virus for risk assessment due to its prevalence in wastewater and its significant public health impact (Wilhelm et al., 2015). There are at least ten genogroups of noroviruses; however, those that infect humans are primarily classified as GI and GII (Parra, 2019). Therefore, this study focused solely on the concentrations of norovirus GI and GII. Risk assessment of all the pathogens was carried out following a four-step QMRA process and the specifics are detailed in (Li et al., 2023). The health risks were computed with and without the consideration of a GDM system applied during the wastewater treatment process. Microbial challenge methods and fluorescence dye tests were performed to develop a reliable approach for ensuring membrane integrity. MS2 bacteriophage was used as a surrogate for the enteric viruses in the experiments to calculate the reduction in virus concentrations. Experiments with a fouled membrane were also conducted to investigate the influence of biofilm on the performance of the water filtration process. Finally, the issues with pore size and pore size distribution of the membrane used for water filtration have been addressed and discussed.

## Materials and Methods

### Overview of WWTP Sampling and Membrane Specifications

Eight wastewater treatment plants were selected to gather waterborne virus concentration measurement data. These included the STEP Vidy WWTP in Lausanne, Switzerland, the ARA Werdhölzli WWTP in Zürich, Switzerland, the Matsushima WWTP in Japan, WWTPs in Los Angeles County Sanitation Districts (LACSD), City of Los Angeles Sanitation and Environment (LASAN), Orange County Sanitation District (OCSD), City of San Diego (SD), and San Francisco Public Utilities Commission (SFPUC) in the USA. Details regarding the enumeration methods for each type of enteric virus in all these WWTPs are introduced in (Li et al., 2023). In all the data analyzed, norovirus GII concentrations consistently exceeded norovirus GI concentrations by approximately one order of magnitude. However, since both genogroups are capable of infecting humans, we used the combined concentrations of both genogroups for further analysis. It is important to note that the norovirus concentrations reported here were determined using molecular methods, which can overestimate the quantity of infectious virus particles (Farkas et al., 2020).

Four types of membranes were used. They were the Microdyn membranes NADIR®US100, NADIR®UP150 from Germany, and the 3 M membranes MicroPES®PH (3 M PH) and MicroPES®EL (3 M EL) from the USA. Information on the pore size of a membrane is often given as nominal pore size, which describes the ability of the membrane to retain 90% of the particles of the rated size or sometimes given as nominal molecular weight cut-off. They are similar concepts, and empirical equations exist to relate these two parameters to each other. These correlations however are to be used with caution since the dimensions of molecules do not only depend on the molecular weight but also on the conformation and properties. Here a method was applied, based on data for Polyvinylidene fluoride and Polytetrafluoroethylene in (Van der Bruggen & Vandecasteele, 2002), which was fitted to provide the following equation:

3 $$y = 220{*}x^{3}$$

where *y* is the molecular weight cut-off in Dalton and *x* is the pore size in nm. The information of all membranes is shown in Table 1.

**Table 1:** Membrane information

Type\parameter	Nominal pore size (µm)	Nominal MWCO (kDa)	Mean bubble point (bar)	Permeability (L/(m^2^hbar)
NADIR®US100	≈ 0.011 (calculated)	100	–	> 100
NADIR®UP150	≈ 0.013 (calculated)	150	–	> 286
MicroPES®PH	0.04	–	2.80	2400
MicroPES®EL	0.1	–	2.06	6000

The ‘–’ sign in the Table indicates that data are not available for those parameters

### Membrane Integrity Tests

For membrane integrity tests, the sensitivity is a very important factor to ensure the regulated requirement of pathogen removal. It refers to the maximum log removal value that can be verified by the test methods reliably. The expression is in terms of a LRV that must be equal to or greater than the required value. For pressure-based tests (i.e., PDT and DAF), the sensitivity can be calculated with the following equation:

4 $${\text{LRV}}\; = \;\log \left( {{\text{VCF}}\; \times \;\frac{{Q_{{\text{P}}} }}{{Q_{{{\text{breach}}}} }}} \right)$$

where *Q*_P_ is the filtrate flow rate, *Q*_breach_ is the flow rate from the breaches corresponding to the smallest response that can be reliably measured, and VCF is a volume concentration factor; for example, the ratio of concentration in the influent and that of the retentate. The sensitivity for surrogate tests is therefore a function of the particle count in the feed and the minimum detectable concentration in the permeate.

#### Microbial Challenge Tests

Microbial challenge tests in drinking water treatment usually employ seeding of representative bacteria, viruses, or oocyst into the feed and then measure the level of these organisms in the permeate (Ferrer et al., 2015). These tests possess obvious advantages, while the surrogates are direct representative of the pathogens relevant to human health and provide high sensitivity and accuracy. In water treatment studies, *Enterococcus faecalis* is commonly used as a bacterial surrogate due to its relevance as a waterborne microorganism and a well-established indicator of fecal contamination. Its ability to survive under environmental stresses, including some disinfection processes, makes it a conservative marker for evaluating treatment efficacy. Additionally, the availability of both pathogenic and non-pathogenic strains allows researchers to model real-world contamination scenarios while maintaining safety in experimental settings (Rivero et al., 2012). As one species of viruses, bacteriophages are ideal surrogates for viruses as they are not directly risky to human, and they are representative of viral pathogen in terms of size, shape, and surface characteristics. Among all species, MS2 is a typical indicator of viruses as it has similar features compared to enteric viruses, which has a diameter of around 30 nm and is one of the smallest viruses existing. Thus, it is of practical importance to measure rejection of MS2 with different membranes (Susan Springthorpe et al., 1993). Apart from the merits mentioned above, both challenge tests with *Enterococcus faecalis* and MS2 are biosafety level one to two thus can be conducted in labs without requiring extensive safety precautions. The plate counting steps for the bacteria are described in the supplemental file and the two-layer plaque counting method for virus is depicted in the supplemental file. All chemicals and reagents used in the experiments were from the Sigma-Aldrich company in the USA.

#### Fluorescent Dye Challenge Tests

Another indirect method for membrane integrity test is the fluorescent dye challenge test. In this method, synthesized particles are stained with fluorescent dye and spiked in the feed (Choi et al., 2011). UV spectrometer and image analysis are applied to detect the fluorescent dye concentration in the permeate and the feed. The *MG1655 E.coli* bacteria were modified to be able to express green fluorescent protein and were used in this experiment as a surrogate for the regular bacteria. Flow cytometer was used to detect the green fluorescent signal in the bacteria using CytoFlex FCM from the Beckman Coulter Company in the USA, with the 488-nm blue laser and the 638-nm red laser.

#### Filtration Setup

**Fig. 1:**
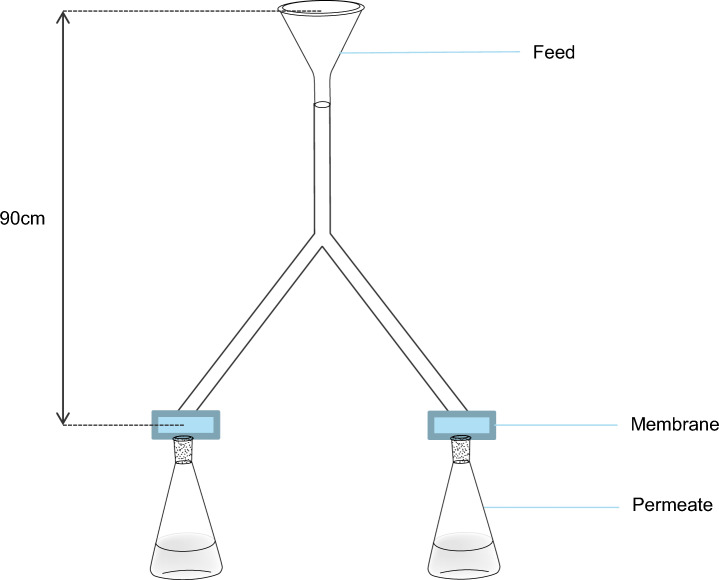
Filtration apparatus for water treatment

In order to determine the membrane rejection of bacteria and viruses, a gravitational filtration setup was used. This setup was constructed using common glassware and plastic tubing, as well as filter holders containing the membrane. Feed water was added to the glass funnel and two Erlenmeyer flasks were used to collect the permeate. The applied hydrostatic pressure was 90 cm water column, corresponding to a pressure of about 90 mbar. Figure 1 shows the filtration apparatus schematically.

### Filtration Setup with Membrane Fouling

**Fig. 2:**
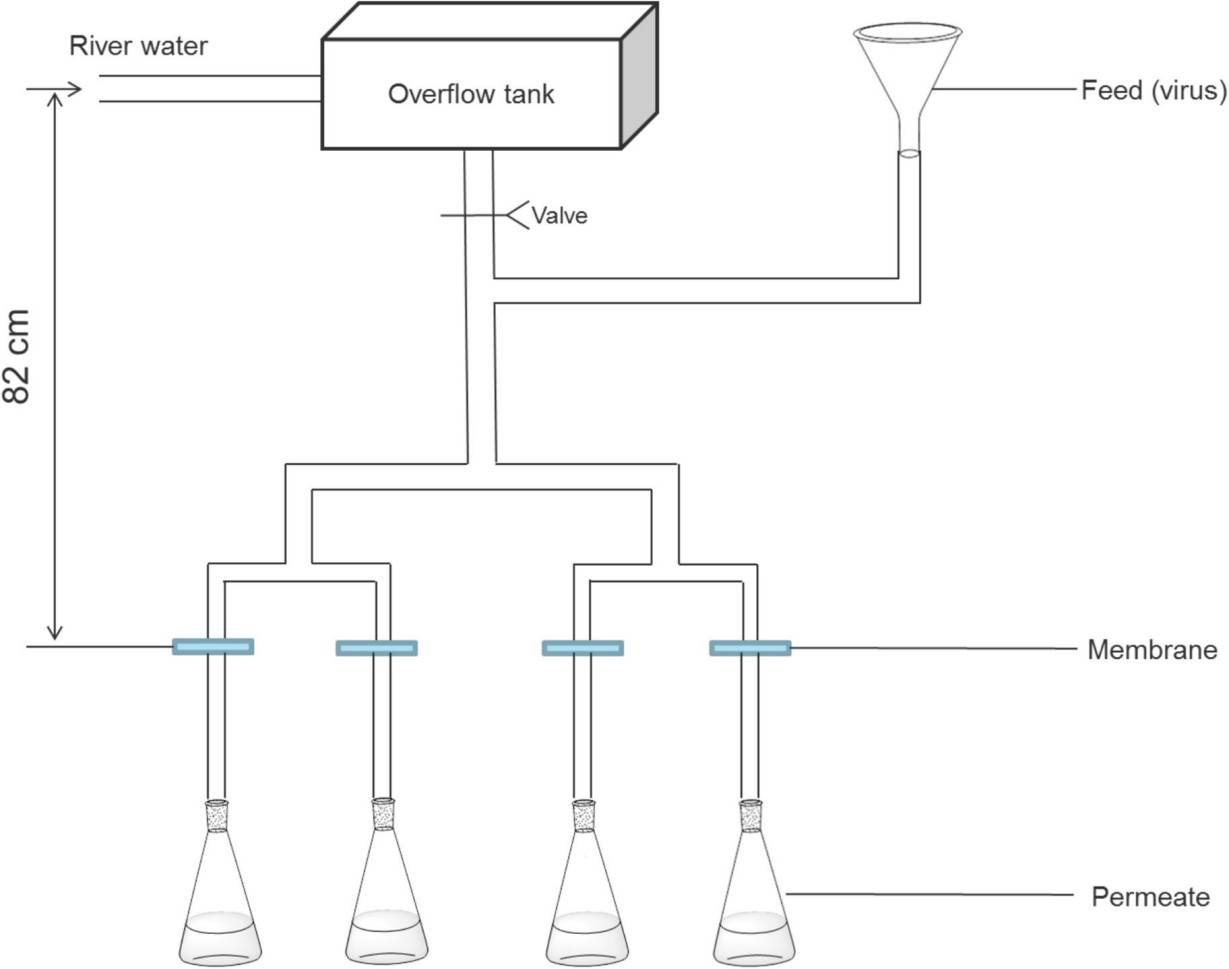
Filtration apparatus for water treatment with biofilm growth

To explore the effect of the fouling layer on the performance of the membrane, especially on the rejection of the MS2 bacteriophage virus, another setup of filtration apparatus was used and the structure is depicted in Fig. 2:

River water was used as feed water and an overflow tank was used to keep a constant pressure head for membrane filtration. Four types of membrane were tested in duplicate of each type in membrane flow cells. The gravitational pressure was kept at 0.082 bar and the temperature was kept at 20–21 °C in the experimental room. When virus rejection tests were conducted, the inflow tubing from the overflow tank was closed and the feed water line was replaced by a funnel containing river water spiked with MS2 bacteriophages at the same gravitational pressure (0.082 bar). Permeate was collected and analyzed according to the assay mentioned above. The tubing on the permeate side was changed each time new permeate was collected to prevent contamination or the effect of regrowth on the permeate side. Flux measurement was conducted with both the river water flow and the feed with virus, and the results from the setup initialization to full biofilm development were recorded. Biofilm growth was visualized and characterized using optical coherence tomography (OCT) at the end of the filtration process.

### QMRA

The QMRA process has four major components: hazard identification, exposure assessment, dose–response assessment, and risk characterization (Fewtrell, 2013). Exposure assessment combines data on the pathogen concentration at the site of interest with information on the rate of intake by an individual. In this study, we assumed that 16 ml of virus contaminated water was taken by one person during each recreational water event in contact with the contaminated water (WHO, 2021). The risk characterization was done by integrating the exposure and dose–response models to estimate (per event) negative health outcomes (infection) as a function of the number of viruses ingested. In this study, a beta-binomial model was chosen for norovirus (Teunis et al., 2020) to evaluate dose–response relations.

## Results

### Microbial Challenge Test

**Fig. 3:**
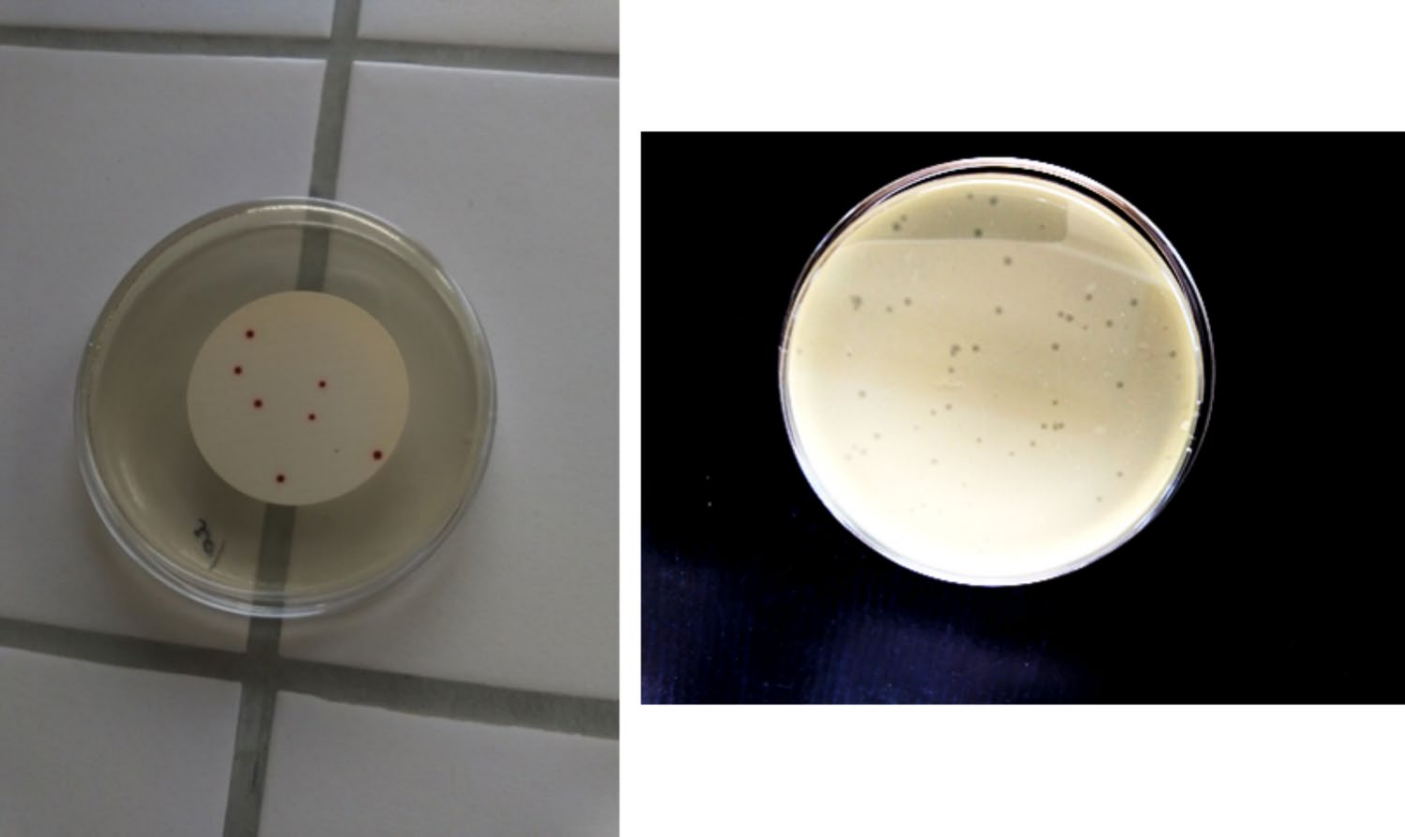
Sample of bacteria colony (left) and MS2 virus plaques (right) on the plate

To obtain bacteria or virus concentrations, Bacteria colonies and virus plaques were identified in each plate and counted for enumeration. Figure 3 demonstrates one sample of the bacteria colony count plate and one virus plaque count plate. Eighteen experiments were conducted with bacteria to evaluate the stability of the membrane integrity test results for membrane integrity test and to compare them with the fluorescent dye method. The autocorrelation coefficient (ACF) and the partial autocorrelation coefficient (PACF) are demonstrated in Figure. S2 in the supplemental file. It could be seen from the results that values of lag orders greater than one all fall in the 95% confidence interval and the values converge to zero as the order of lag increases.

### Green Fluorescent Bacteria Test

**Fig. 4:**
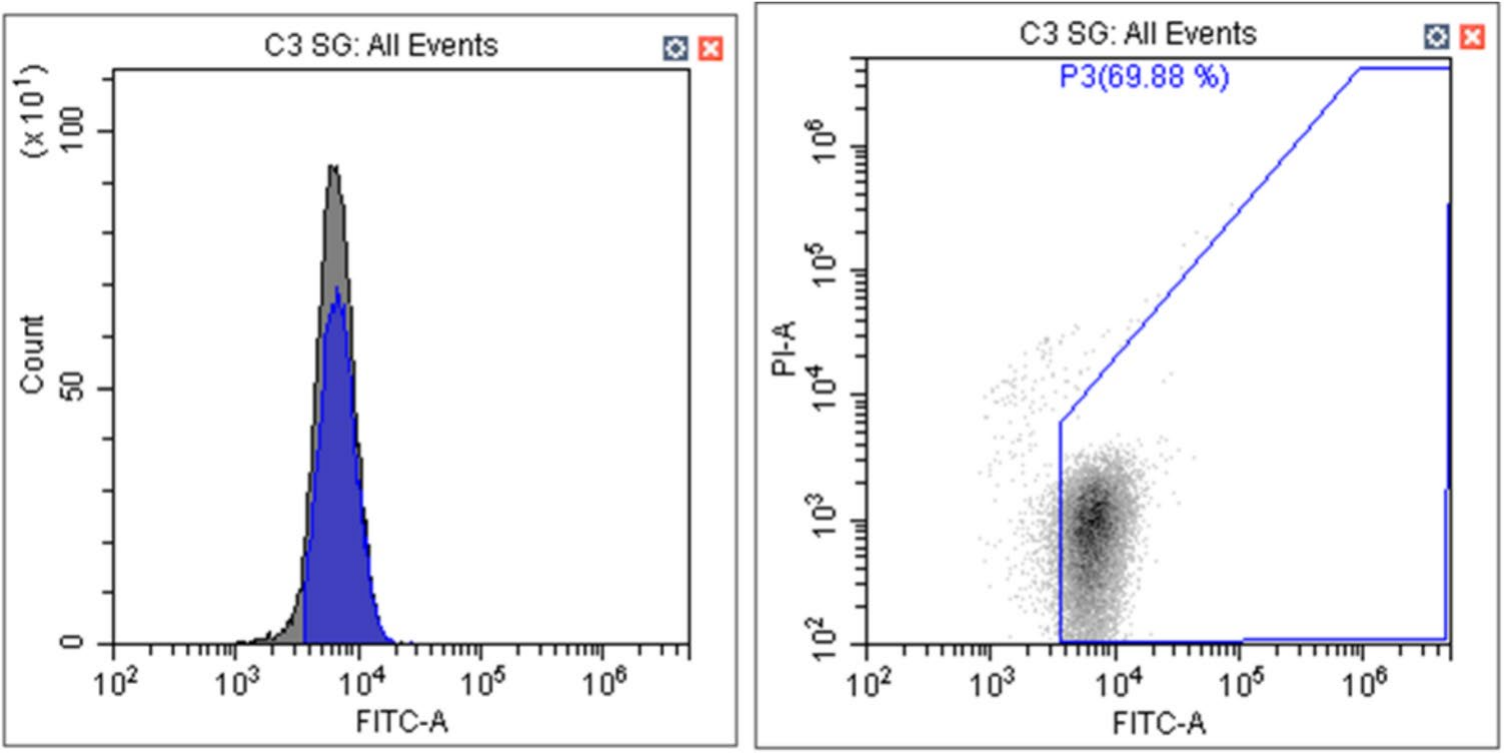
Green fluorescent bacteria tests from flow cytometry

The green fluorescent bacteria which contain the green fluorescent protein were used to verify if this alternative could be used properly in the membrane integrity test. Figure 4 shows the result acquired from the flow cytometer. On average, the concentration of bacteria was 209 cells/µl of sample. This number was lower than that obtained through microbial cultivation, differing by a factor of approximately 1.5, with the cultivation method measuring 320 cells/µl per sample. However, this method can only be applied to bacteria, not viruses.

### Log Removal of Bacteria

**Fig. 5:**
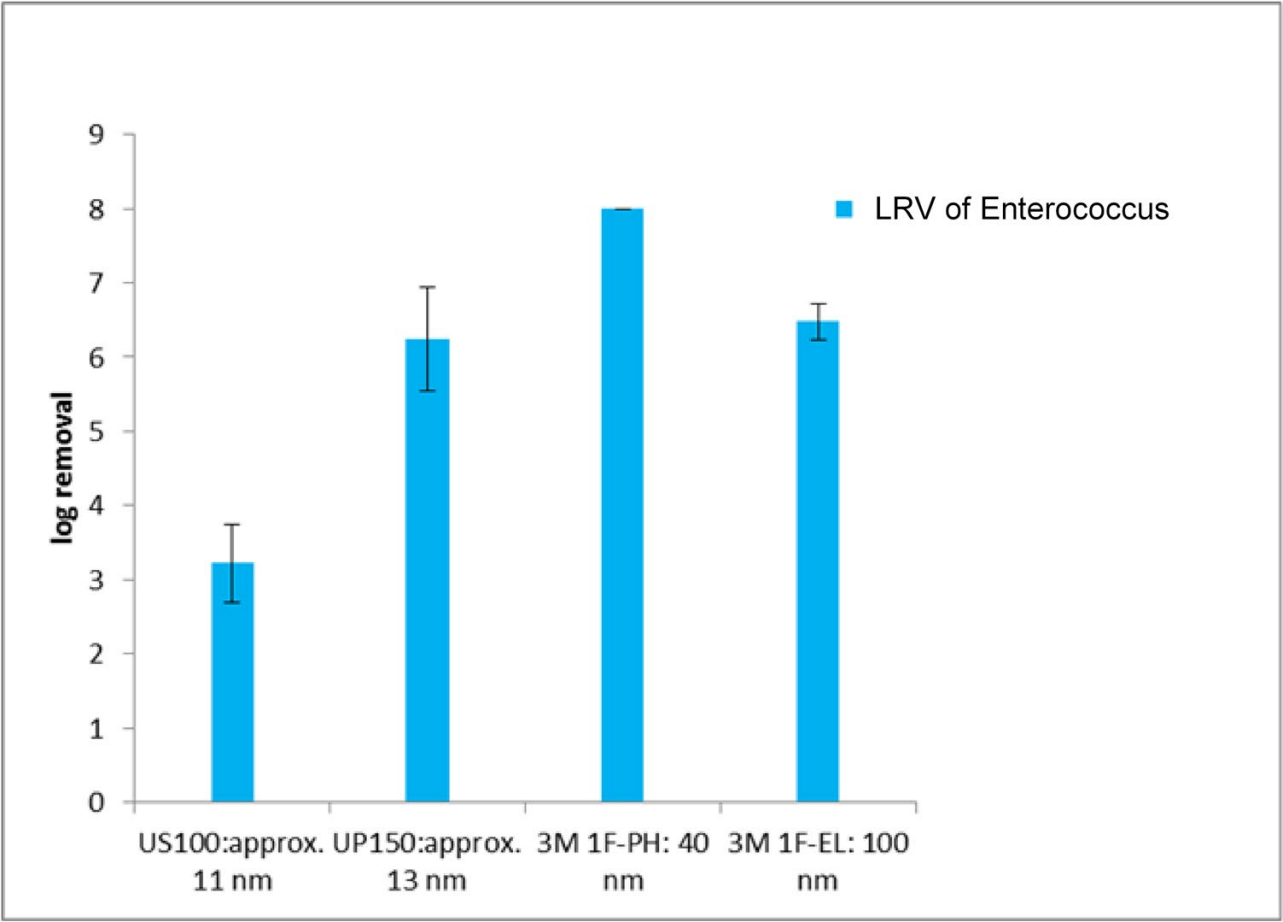
LRV (in CFU/ml) of bacteria for 4 types of membranes

The measurement of bacteria rejection was conducted once a week for 4 weeks using the same membrane and each sample was analyzed using the bacteria colony counting method. Four types of membranes were tested and an average log removal value for all membranes was calculated. Figure 5 shows the LRV of *Enterococcus* bacteria for all four membranes used in this study and the results are expressed in log colony-forming units per milliliter (CFU/ml). The highest removal was 8 logs observed for the 3MPH membrane with a nominal pore size of 40 nm, while the lowest removal was 3.4 logs recorded for the US100 membrane with a nominal pore size of about 11 nm, indicating that the membrane was not dense. The mean removal rate was 6.2 logs.

### Log Removal of Virus

**Fig. 6:**
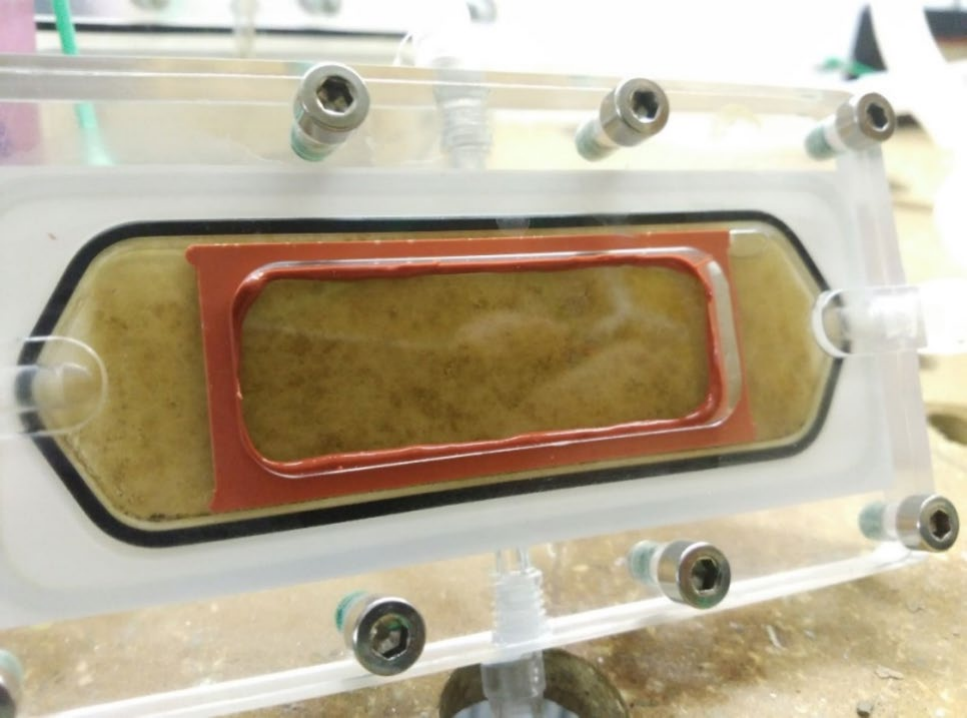
Flowcell with biofilm growth

**Fig. 7:**
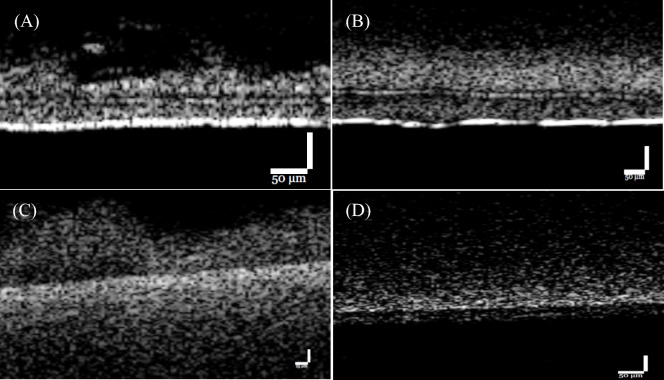
OCT image of fouled membranes. **A** US100, **B** UP150, **C** 3 M PH, and **D** 3 M EL

For the study of virus, the removal of MS2 bacteriophage was evaluated with a clean membrane as well as with a fouled membrane. A rejection test was done with a clean membrane and after 3 weeks of operation. A biofilm layer is clearly visible after 3 weeks of operation, when fed with river water, as shown in Fig. 6. The OCT images in Fig. 7 demonstrate the thickness and the profile of the biofilm grown on the membrane. It could be seen from the OCT measurements that for US100 and UP150 membranes, biofilm growth was much more noticeable than on the other two membranes and the biofilms were much thicker and denser.

**Fig. 8:**
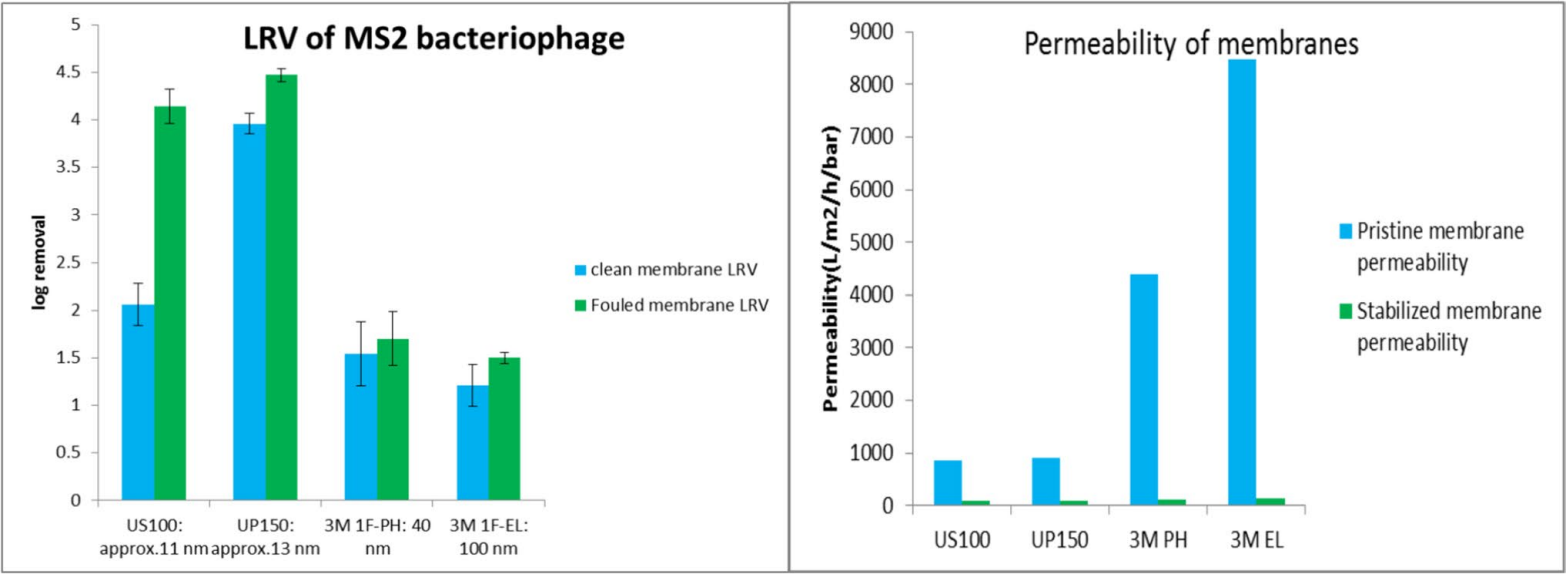
LRV (in PFU/µl) of MS2 and permeability for clean membrane and with biofilm growth

Biofilm formation on the membrane surface not only alters the performance of membrane in terms of log removal of pathogens but also changes the permeability of the membrane. Figure 8 depicts the LRV of MS2 and the permeability of the clean and fouled membranes with biofilm growth. The MS2 results are expressed in log plaque-forming units per microliter (PFU/µl). The highest log removal for MS2 with clean membranes was 4 logs, seen for the UP150 membrane with a nominal pore size of about 13 nm, while the lowest removal was 1.21 logs for the 3MEL membrane, with a pore size of 100 nm. The average removal was 2.3 logs. When biofilm growth took place on the membrane, the removal of MS2 virus was observed for all four membranes. The most significant increase was observed with the US100 membrane, which was less dense. This led to an increase in log removal from 2.1 to 4.1. The highest log removal for MS2 with fouled membranes have reached 4.5. However, large decrease in membrane permeability was also noticed for all types of membranes, dropping to around 100 L/m^2^/h/bar for all membranes, from at least 733 L/m^2^/h/bar. The decrease of permeability under 0.9 bar of operational water head was dramatic and almost led to an equivalent final permeability for all four membranes.

### Risk Assessment of Norovirus With and Without Membrane Filtration

**Fig. 9:**
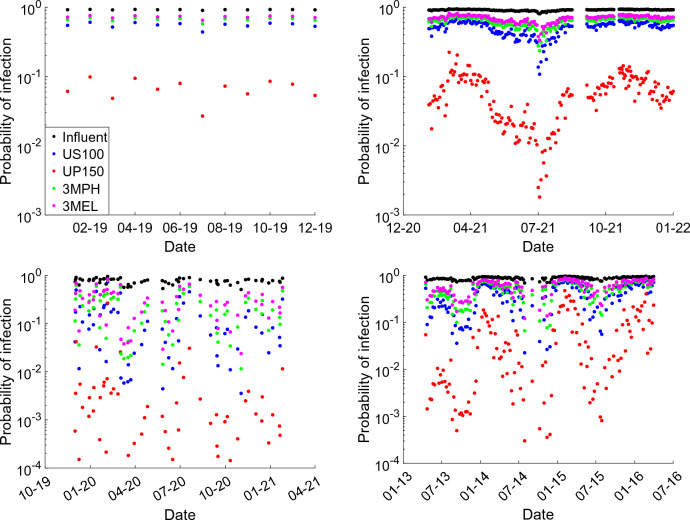
Infection risks of norovirus of WWTP influents and the treated wastewater including GDM system with clean membrane based on the water sample data in Lausanne Switzerland, Zürich Switzerland, Matsushima, Japan, and in the USA. The date in the x-axis indicates the month and the year of the time of sampling

**Fig. 10:**
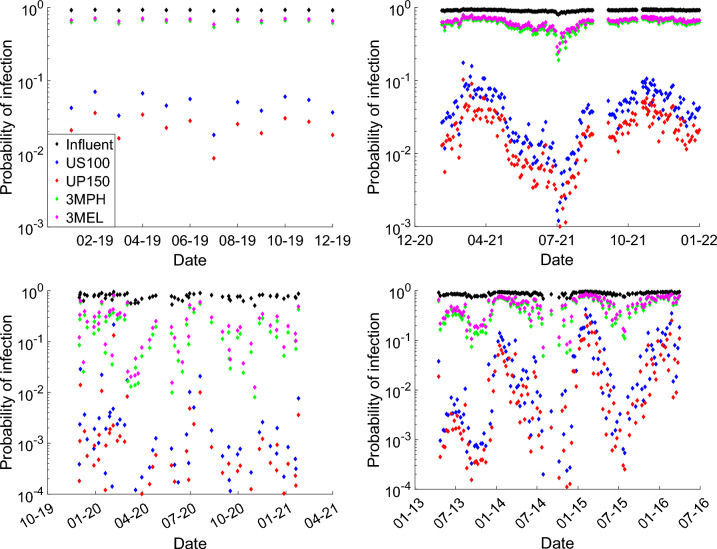
Infection risks of norovirus of WWTP influents and the treated wastewater including GDM system with fouled membrane based on the water sample data in Lausanne Switzerland, Zürich Switzerland, Matsushima, Japan, and the USA. The date in the x-axis indicates the month and the year of the time of sampling

Norovirus infection risks were assessed based on three scenarios: influent concentrations, concentrations after WWTP treatment with additional gravity-driven membrane (GDM) filtration, and concentrations after WWTP treatment with additional GDM filtration under membrane fouling conditions. Figure 9 demonstrates the infection risks for raw wastewater influent and for effluent treated with WWTP and GDM filtration. Infection risks for the raw influent were consistently near 100% or over 95%, emphasizing the extreme danger of norovirus in untreated wastewater and the necessity of proper treatment before discharge into natural water bodies. Among the tested membranes, the UP150 membrane exhibited the most significant reduction in infection risk, lowering risks to below 0.02% compared to the > 95% risks of the raw influent. The US100 membrane provided the second highest reduction in virus rejection, while the least reduction was observed for the 3MEL membrane, which has the largest nominal pore size. When membrane fouling occurred during the filtration process, the virus removal rates showed variability. As illustrated in Fig. 10, the lowest risks achieved with the UP150 membrane under fouled conditions remained largely unchanged. However, for the US100 membrane, the infection risk dropped significantly under fouling, with values approaching those achieved by the UP150 membrane. This additional virus removal effect under fouled conditions was less pronounced for the 3MPH and 3MEL membranes, both of which have larger nominal pore sizes. These findings highlight the complex interplay between membrane fouling and virus rejection efficiency, with fouling occasionally enhancing performance for certain membranes while having limited impact on others.

## Discussion

### Membrane Integrity Test

Microbial challenge tests with both bacteria and viruses were conducted to evaluate membrane integrity. To assess the stability and reproducibility of the agar plate method used in bacteria challenge tests, statistical analyses were applied to the data series. As shown in Figure S1, the data series demonstrates consistent results across different experiments, indicating strong stability. Statistically, data series are considered stable over time if the autocorrelation function (ACF) and partial autocorrelation function (PACF) values of lag orders larger than 1 fall within the 95% confidence interval and converge to zero as the lag order increases. Figure S2 confirms this, as the values converge to zero with increasing lag order and remain almost entirely within the confidence interval. These findings confirm that the data series is stable over time, thereby justifying the reproducibility of the experiments and the reliability of the microbial challenge method for membrane integrity testing. The microbial challenge tests possess obvious advantages as the surrogates are direct representative of the pathogens in concern to human health and provide high sensitivity and accuracy (Antony et al., 2014). While the microbial challenge test is highly accurate and sensitive, it is also time-consuming and impractical for real-time online monitoring. In contrast, the fluorescent dye challenge test offers a rapid alternative and can be easily integrated into an online monitoring system. However, as observed in the results, the bacterial counts obtained from the flow cytometer were lower than those from the microbial cultivation method. This discrepancy suggests that while the green fluorescent protein signal is detected effectively by the flow cytometer, factors such as cell aggregation or shading effects may impact the accuracy of the measurements. Further research is required to fully validate the use of green fluorescent bacteria for membrane integrity testing in GDM systems. Nevertheless, this approach holds significant promise due to its speed, sensitivity, and compatibility with automated and online monitoring setups, aligning well with the key criteria outlined in (Farahbakhsh et al., 2003) for selecting an ideal membrane integrity monitoring technique.

### Removal of Bacteria, Virus, and the Relation with Membrane Pore Sizes

As indicated in Fig. 5, all four membranes tested achieved a removal rate greater than 99.9% for *Enterococcus faecalis*, which has a size of approximately 1 µm. Given that the nominal pore sizes of all membranes are smaller than 1 µm, this high rejection rate aligns with expectations. Earlier studies on membrane filtration of bacteria also suggested high retention rates, ranging from 5 logs to even complete removal (Hai et al., 2014). However, the rejection rates for MS2 virus were lower for all membranes compared to bacteria, which is consistent with the smaller size of MS2. The 3MPH and 3MEL membranes were not expected to retain MS2 due to the large pore size of these membranes. The high removal rate found with the UP150 is closely comparable to other studies concerning removal of MS2 with ultrafiltration membranes (Boudaud et al., 2012; ElHadidy et al., 2013). The experimental results show variability in performance between membranes. For example, the US100 membrane (nominal pore size ~ 11 nm) achieved a log removal value (LRV) of 3.4, while the 3MPH membrane (nominal pore size ~ 40 nm) achieved the highest LRV of 8. This indicates that the membrane with the smallest nominal pore size (US100) exhibited the lowest rejection rate, while the 3MPH membrane, with the second largest nominal pore size, demonstrated the best performance. Similarly, as shown in Fig. 8, the US100 membrane exhibited lower MS2 rejection than the UP150 membrane, despite its nominal pore size being smaller (11 nm vs. 13 nm). These results highlight a key limitation in the interpretation of nominal pore size. The nominal pore size of a membrane reflects its ability to retain 90% of particles at or larger than the specified size but does not provide information about the retention rates of particles smaller or larger than the rated size. The experimental results suggest that factors beyond nominal pore size, such as pore size distribution and structural properties of the membrane, significantly influence particle rejection. The US100 membrane may had a broader pore size distribution or larger effective pores, reducing its virus retention despite the nominal rating, while the UP150 membrane likely had a sharper pore size cut-off than the US100 membrane, leading to higher rejection rates despite their nominal pore sizes being larger. Moreover, particle rejection is not solely determined by physical pore size. Antony et al. found that the physical and chemical properties of the membrane, feedwater, and pathogen surface may play a role in the removal process (Antony et al., 2012). It was also found that the pressure applied on the membrane can significantly affect the rejection of waterborne pathogens (Arkhangelsky & Gitis, 2008). Other studies have shown that the configuration of the filtration apparatus can influence the removal process as well (Long et al., 1981). These findings underscore that nominal pore size alone provides only a general indication of pathogen removal efficiency. A more detailed characterization of membrane pore size distribution and other membrane properties is essential for accurately predicting the removal rates of specific pathogens.

### Risk Assessment of Norovirus Before and After Treatment with the GDM System and the Influence of Membrane Fouling

Norovirus concentration in wastewater is highly variable and can be as high as 10^6^ GC/ml in many cases, although it should be noticed that measurements by molecular methods may overestimate the infectious concentration. As indicated in Fig. 9, infection risks for raw wastewater influent were all over 95%, implying that norovirus concentration in raw wastewater is too high and people are mostly surely infected if they are exposed to such contaminated water. Traditional wastewater treatment processes typically reduce enteric virus concentration by 1–3 logs and it is not assured to lower the risk to a safe level (Kitajima et al., 2014). The additional treatment by GDM here shows that the most significant decrease in infection risks could be below 0.02% and this is more than four magnitudes lower than the original risk. Here, the UP150 membrane which has a nominal pore size of 13 nm demonstrated the best performance for virus removal and reduction for infection risks. The average reduction in infection risk for this membrane was 2.1 logs. The US100 membrane displayed the second-best reduction in virus rejection, while the least reduction was observed for the 3MEL membrane with the largest pore size.

However, when membrane fouling happens and biofilm grows, the virus removal rate has been altered for the four membranes tested, so was the infection risks. The OCT images in Fig. 7 demonstrated that the thicknesses of biofilms grown on the four membranes are different. The US100 and UP150 membranes have thicker and denser biofilm layers, which may explain why the drop of virus concentration is higher in the experiment setups with these two membranes compared with the rest. As seen in Fig. 10, while for other three membranes the changes in virus removal and thus the risk levels are moderate or minor, for US100 membrane the risk levels have dropped significantly, the lowest values are very close to the UP150 membrane extremes with the best performance in virus rejection. The average infection risk reduction with a fouled membrane was 2.54 logs, this value is even 0.44 logs better than the reduction with a clean membrane, indicating that biofilm growth indeed elevated virus removal compared with clean membranes. Other studies also found positive correlation between virus removal and membrane fouling (Zhang et al., 2022). Nonetheless, the relative performance of all four membranes remains consistent, with the UP150 showing the best performance and the 3MEL type performing the worst. However, as indicated by the permeability values in Fig. 10, the flow flux has significantly decreased due to membrane fouling. In the GDM process, membrane fouling is not always undesired, as the flux stabilizes due to the fouling layer and more importantly the presence of a biofilm can also contribute to the additional removal of pathogens, acting as a “secondary membrane,” which is modifiable in certain cases (Ma et al., 2001). The cost is, however, the drop-in membrane permeability. Thus, the management of membranes in GDM (Gravity-Driven Membrane) systems to control membrane fouling and biofilm growth becomes highly complex, requiring careful monitoring and maintenance strategies. Effective control measures are essential to mitigate these challenges, but the precise mechanisms of fouling and biofilm formation in such systems remain insufficiently understood. Therefore, further investigation is needed to develop more comprehensive strategies and provide clearer guidance for operational plans, ensuring that these systems remain cost-effective and sustainable over time.

## Conclusion

Pathogens such as bacteria and viruses in wastewater are often overlooked compared to other treatment parameters, despite their significant threat to public health. In this study, we investigated the effectiveness of a gravity-driven membrane (GDM) system in enhancing pathogen removal and reducing infection risks in wastewater effluent. The results showed that GDM systems can significantly lower pathogen concentrations, achieving up to 8-log reduction for bacteria and 4.5-log reduction for viruses. The infection risk from the waterborne virus, norovirus, was reduced by approximately 4 logs following GDM treatment. Membrane integrity was found to be critical for maintaining optimal pathogen removal performance. Both microbial challenge tests and fluorescent dye tests proved effective for assessing membrane integrity, with the former offering higher sensitivity and accuracy, and the latter providing a quicker, automated alternative. While membrane pore size plays a key role in pathogen rejection, our study revealed that nominal pore size alone does not fully predict removal efficiency. The membrane pore size distribution significantly influences the overall rejection rate, making it an important factor to consider in pathogen removal calculations. Membrane fouling, often considered undesirable, was shown to have a surprising benefit in some cases. Biofilm growth on fouled membranes acted as a “second membrane,” enhancing virus removal by up to 2 additional logs. However, this increased pathogen rejection came at the cost of reduced membrane permeability, highlighting a trade-off between improved pathogen removal and system efficiency. This study underscores the importance of membrane maintenance, pore size distribution, and proper monitoring in optimizing GDM systems for pathogen removal and minimizing public health risks.

## Supplementary Information

Below is the link to the electronic supplementary material.

Supplementary file1 (DOCX 35 KB)

## Data Availability

No datasets were generated or analysed during the current study.
