# Supplementary material for: Evaluating the Performance of Gravity-Driven Membrane Filtration for Waterborne Pathogen Removal and Public Health Protection
Source: Food Environ Virol. 2025 Jul 14;17(3):40. doi: 10.1007/s12560-025-09655-1 (PMC12259810; doi:10.1007/s12560-025-09655-1)
Supplement: Supplementary file 1 — Supplementary file1 (DOCX 35 KB) [file 12560_2025_9655_MOESM1_ESM.docx]

## Supplemental document

Evaluating the performance of gravity-driven membrane filtration for waterborne pathogen removal and public health protection

# Chaojie Li^*^

Department Surface Waters Research & Management, Eawag, Swiss Federal Institute of Aquatic Science and Technology, Seestrasse 79, 6047, Kastanienbaum, Switzerland

^*^ Corresponding author:

Chaojie Li: chaojie.li@eawag.ch

## Bacterial rejection test

- Preparation of agar media

Add 42g Enterococcus selective agar into 1L nanopure water and stir, heat to boil. Pour the solution on petri dishes as soon as possible, when the agar is set, bag the plates and store in the fridge for up to one month.

- Prepare the TBS (Tris Buffer Saline)

Dissolve 6.05g Tris and 8.76g NaCl in 800ml of water.

Adjust PH to 7.6 with 1M HCl. (can be a bit over 7.6 but not less)

Add water to reach the volume of 1L

It is stable at 4 C° for 3 months.

- Conditioning of bacteria

Pipet 1ml of defrosted Enterococcus faecalis in a tube with TBS filled up to 50ml and put it in the Incubator at 35 C° and rotate at 120 rad/min overnight (18 hours).

- Preparation of general test water

Add 0.1ml tannic acid, 2ml sea salt water and 2ml sodium bicarbonate to 1L Schott bottle with nanopure water filled up to 1L line and add 1ml of the enterococci solution.

- Filtration and bacteria collection

Make dilutions to 10^-4^ and 10^-5^, filtrate with a vacuum filtration device from UMIK, 100ml per membrane filter, white side up (no grid side) and attach the permeate side of the filter to the agar plate.

- Incubation and enumeration

Put agar plate with filters attached into the incubator and let it grow overnight at 35 C°. Count the colonies on the plates next day.

## Virus rejection test

1. Preparation of media

Various media for this bacteriophage needs to be prepared before the analysis of MS2 plague counting. The necessary media include:

1.1 Nutrient Broth Number 1 (NB1)

- Add 25g NB1 powder (Sigma**^®^**) to 1 L nanopure water and distribute in suitable size Schott bottles.
- Autoclave and store for up to 1 month.

1.2 Nutrient Agar Number 1 (NA1 Agar plate)

- Add 25g NB1 powder to 1 L nanopure water and mix well.
- Add 14g Bacteriological Agar and mix well.
- Decant into 1 L Schott bottle and autoclave.
- After autoclaving incubate at 60 C° for 1 hour.
- Per liter, add 10ml of Glucose: calcium chloride solution.
- Pour thin layer into Petri dishes and leave to set for around 1 hour, invert, bag and store in the fridge.

1.3 Semi Solid Nutrient Agar Number 1 (ssNA1 )

- Add 25g NB1 powder to 1 L of nanopure water and mix well.
- Add 7g Bacteriological Agar and mix well.
- Decant into Schott bottles
- Autoclave and store for up to 1 month

1.4 Glucose: calcium chloride solution

- Add 3g calcium chloride dehydrate, 10g glucose in 1 L nanopure water and mix well.

2. Analysis

One day before analysis

- Remove vial of E.coli DSM8589 from freezer.
- When thawed, add to 10 ml NB1 in sterile test tube and incubate overnight at 37 C°.

On the day of analysis

- Take 1 ml of the overnight culture and add to 9 ml NB1.
- Incubate at 37 C° and occasionally measure OD at 600nm using NB1 as blank.
- When OD reaches 0.5, the inoculum culture is ready for use (between 2-4 hours). The culture should either be used immediately or placed on ice until use.
- Melt ssNA1 using microwave.
- Per 100 ml of ssNA1, add 1 ml of glucose: calcium chloride solution.
- Distribute 2.5 ml aliquots of ssNA1 to test tubes and place in water bath at 50 C°.
- Take 3-4 test tubes containing ssNA1 at a time and add 100 µl of sample to be tested to each tube and 1 ml of inoculum culture to each tube.
- Mix well using vortex and pour onto NA1 agar plates which is the first layer.
- Repeat above two stages until all samples have been processed.
- Allow Petri dishes to solidify, once done, invert and incubate at 37 C° for 18h and count.

**Figure. S1** Feed concentration measurement from all experiments

**Figure. S2** ACF and PACF of the measurement time series
